# Ribonuclease Inhibitor 1 (RNH1) Regulates Sperm tsRNA Generation for Paternal Inheritance through Interacting with Angiogenin in the Caput Epididymis

**DOI:** 10.3390/antiox13081020

**Published:** 2024-08-22

**Authors:** Zhuoyao Ma, Ningyuan Tang, Ruiyan Zhang, Hanyu Deng, Kexin Chen, Yue Liu, Zhide Ding

**Affiliations:** 1 Department of Histology, Embryology, Genetics and Developmental Biology, Shanghai Key Laboratory for Reproductive Medicine, Shanghai Jiao Tong University School of Medicine, Shanghai 200025, China; s82821515@sjtu.edu.cn (Z.M.); lester-tang@sjtu.edu.cn (N.T.); 2 Department of Teaching Laboratory Center for Basic Medicine, Chengdu Medical College, Chengdu 610500, China; 3 Department of Clinical Medicine, Shanghai Jiao Tong University School of Medicine, Shanghai 200025, China; ruirui0650@sjtu.edu.cn (R.Z.); calliope@sjtu.edu.cn (H.D.); cjk123@sjtu.edu.cn (K.C.)

**Keywords:** ribonuclease inhibitor 1 (RNH1), angiogenin (ANG), tRNA-derived small RNAs (tsRNAs), environmental stresses, epigenetics

## Abstract

Environmental stressors can induce paternal epigenetic modifications that are a key determinant of the intergenerational inheritance of acquired phenotypes in mammals. Some of them can affect phenotypic expression through inducing changes in tRNA-derived small RNAs (tsRNAs), which modify paternal epigenetic regulation in sperm. However, it is unclear how these stressors can affect changes in the expression levels of tsRNAs and their related endonucleases in the male reproductive organs. We found that Ribonuclease inhibitor 1 (RNH1), an oxidation responder, interacts with ANG to regulate sperm tsRNA generation in the mouse caput epididymis. On the other hand, inflammation and oxidative stress induced by either lipopolysaccharide (LPS) or palmitate (PA) treatments weakened the RNH1-ANG interaction in the epididymal epithelial cells (EEC). Accordingly, ANG translocation increased from the nucleus to the cytoplasm, which led to ANG upregulation and increases in cytoplasmic tsRNA expression levels. In conclusion, as an antioxidant, RNH1 regulates tsRNA generation through targeting ANG in the mouse caput epididymis. Moreover, the tsRNA is an epigenetic factor in sperm that modulates paternal inheritance in offspring via the fertilization process.

## 1. Introduction

Over the past decades, intergenerational inheritance caused by environmental factors has attracted increasing interest in the field of epigenetic research [1]. Recent studies showed that environmental factors, such as mental stress, drugs, and unhealthy eating habits, can compromise human health. Moreover, the environmental factor-induced acquired traits can be inherited between generations, and even transmitted across several generations [2,3,4]. To probe for the reasons of these biological effects, many studies showed that environmental exposure, lifestyle, and health status can change the expression profiles of sperm small non-coding RNAs (sncRNAs). They mainly include tRNA-derived small RNAs (tsRNAs), rRNA-derived small RNAs (rsRNAs), and microRNAs (miRNAs) [5,6,7]. These altered non-coding RNAs can be transmitted from the sperm through fertilization into the offspring and then modify the regulation of some genes in the zygote. Such interaction may finally result in the expression of acquired traits by the offspring induced by environmental stresses [7]. For instance, among these non-coding RNAs, tsRNAs mediate this process in sperm that accounts for paternal epigenetic inheritance [8].

Mammalian spermatozoa released from the testis extrude most of their cytoplasm, which nearly terminates transcription. In other words, either the cytoplasmic storage capacity or any transcriptional capability can blunt the results of environmental changes [9,10]. On the other hand, the epididymis is a critical organ for sperm functional maturation, which is dependent on the interactions between the sperm and epididymal epithelial cells mainly lining the lumen of the segment between the caput and the corpus epididymis [11,12]. During the epididymal transit, spermatozoa receive additional proteins and non-coding RNAs from the epididymal lumen through crosstalk with the epididymosomes, a type of exosome-like extracellular vesicle generated by epididymal epithelial cells [13,14]. On the other hand, tsRNA expression is significantly increased and exosomes have been reported to transfer tsRNAs into the sperm [11]. Furthermore, it was recently reported that environmental changes can indirectly induce alteration of tsRNA expression levels in the sperm, and angiogenin (ANG) in the mouse caput epididymis regulates sperm tsRNAs and mediates paternal inflammation-induced metabolic disorders in the offspring [15].

RNH1, also named ribonuclease inhibitor 1, is generally expressed in human tissues [16]. This protein contains cytoplasmic leucine-rich repeats, which bind to tryptic-type ribonuclease with femtomolar affinity, rendering it inactive [16]. Several studies validated that RNH1 interacts with ANG and prevents tsRNA generation, which acts as an oxidation and inflammation sensor in cells [17,18]. In addition, RNH1 regulates some miRNAs, such as miR-21, miR-99a, miR-99b, and miR-101, in various different cell types [19,20]. Thus, these considerations prompted us to determine whether the RNH1 expression is related to changes in the tsRNA expression level in the epididymis, and, moreover, what the role of RNH1 is in sperm maturation.

In the current study, we initially identified the interaction between RNH1 and ANG in the mouse caput epididymis, and then demonstrated that inflammation and oxidative stress do indeed weaken this interaction, which upregulates ANG-induced tsRNA expression levels.

## 2. Materials and Methods

### 2.1. Animals

All animal experiments were conducted in accordance with the International Guidelines for the Protection of Animals Involved in Biomedical Research, and the experimental protocols were approved by the Ethics Committee of the Shanghai Jiao Tong University School of Medicine (No. A2019-029 and A2022-048). All mice were housed under pathogen-free conditions with free access to water and food, temperature and humidity control, and constant light and dark cycles at the Animal Center of Shanghai Jiao Tong University School of Medicine.

### 2.2. Cell Culture

For primary culture of epididymal epithelial cells (EEC), the caput epididymis was isolated from 3-week-old male mice, and then in the cell culture laboratory, the caput epididymis was cut with eye scissors in sterile PBS containing penicillin and streptomycin. The treated tissues were dispersed in PBS containing type IV collagenase (2 mg/mL)/DNase (0.5 mg/mL) for 40 min at 34 °C. After PBS washing and gravimetric precipitation, additional digestion steps were performed with accutase cell dissociation reagent (Innovative Cell Technologies, San Diego, CA, USA) at 34 °C for 15 min. Finally, cells were collected and washed with PBS by centrifugation at 1000× *g* for 5 min. Epithelial cells were then purified by differential adhesion. The purity of primary epididymal epithelial cells was routinely analyzed by immunofluorescent staining with cytokeratin 8 (CK8) (a marker for epithelial cells) and vimentin (a marker for fibroblasts as negative control) [21]. In our previous work, we documented the results of immunofluorescent staining with cytokeratin 8 and vimentin in EEC [22]. EEC were cultured in Iscove’s Modified Dulbecco’s Medium (Gibco, New York, NY, USA) supplemented with 10% fetal bovine serum (FBS) (Gibco) and 1 nM 5α-DHT (Sigma-Aldrich, St. Louis, MO, USA), at 34 °C, 5% CO_2_.

The cell lines 3T3 were obtained from National Collection of Authenticated Cell Cultures and cultured in Dulbecco’s Modified Eagle Medium (Gibco) containing 10% FBS, at 37 °C, 5% CO_2_. The 3T3 cell line, also named NIH 3T3, is a fibroblast-like cell line, which is initially isolated from mouse embryonic tissue. This cell line has become an important tool widely used in the research field of cell biology and molecular biology due to its growth characteristics and genetic stability [23,24]. The mouse proximal caput epididymis-1 (PC1) cell line, a pure population of epithelium-derived caput principal cells, is generally employed in the study of mammalian epididymal function [25,26], which was a gift obtained from Dr. Qiang Liu. The PC1 cell line was cultured in Iscove’s Modified Dulbecco’s Medium (Gibco) supplemented with 10% fetal bovine serum (FBS) (Gibco) and 1 nM 5α-DHT (Sigma-Aldrich), at 34 °C, 5% CO_2_.

### 2.3. Western Blot Analysis

Tissues or cells were homogenized in an ice bath in a Nuclear and Cytoplasmic Protein Extraction Kit (Yeasen, Shanghai, China) containing a protease inhibitor mixture (Roche, Mannheim, Germany), and proteins were extracted as described [27]. Protein concentrations were then determined using a BCA protein assay kit (Thermo Fisher Scientific, Rockford, IL, USA).

Protein samples (20 µg) were separated using 8–15% denaturing polyacrylamide gels and then transferred to polyvinylidene difluoride membranes (Millipore, Billerica, MA, USA). The membranes were blocked with 5% bovine serum albumin (BSA) and then incubated overnight at 4 °C with primary antibodies against RNH1 (1:2000 dilution, Abcam, Cambridge, UK), ANG (1:2000 dilution, Proteintech, Wuhan, China), GAPDH (1:5000 dilution, Proteintech), and β-actin (1:5000 dilution, Abcam), respectively, followed by incubation with primary antibodies relevant secondary antibodies (1:2000 dilution, Proteintech). Signals were generated by enhanced chemiluminescence (Millipore) and detected with a luminescence image analyzer (GE Imagination LAS 4000, GE imagination at work, Barrington, IL, USA). Finally, the results of protein band intensity were quantified with ImageJ (USA) software version 1.54j.

### 2.4. Immunofluorescence (IF) Analysis

EEC were cultured on glass-bottom Petri dishes (Cellvis, Mountain View, CA, USA). Slides or culture dishes were then fixed with 20% paraformaldehyde for 4 min at 4 °C. Non-specific binding sites were blocked with 10% BSA/PBS for 60 min at room temperature, followed by permeabilization treatment with 0.1% TritonX-100 for 10 min. Sections were incubated with RNH1 antibody (1:200 dilution, Santa Cruz Biotechnology, Dallas, TX, USA) and/or ANG antibody (1:200 dilution, Proteintech), respectively, overnight at 4 °C. Meanwhile, normal rabbit IgG or normal mouse IgG was used as the negative control. After washing with PBS three times, then the fluorescently labeled secondary antibodies (Alexa Fluor 488 AffiniPure Goat Anti-Rabbit IgG (H + L) or Alexa Fluor 594 AffiniPure Goat Anti-Mouse IgG (H + L), 1:200 dilution; Yeasen) were employed and incubated with sections for 2 h, while cell nuclei were re-stained with DAPI (Sigma-Aldrich). Fluorescence signals were detected under a laser scanning confocal microscope (Carl Zeiss LSM-8, Oberkochen, Germany) equipped with an argon laser (510 nm), a helium/neon laser (488 nm), an EC Plan-NEOFLUAR 543×/63.1 objective, and a LD LCI Plan-APOCHROMAT 25×/25.0 objective (Zeiss, Oberkochen, Germany). Digital images were taken and processed using AIM software Ver. 3.63.15 (Zeiss Systems). The strength of co-localization was quantified by ImageJ (USA) software.

### 2.5. Co-Immunoprecipitation

Protein samples were washed with PBS and then harvested and lysed directly in RIPA lysis buffer. Lysates were incubated with beads (Yeasen) with rotation at 4 °C overnight. Then RNH1 (Abcam) was added with rotation at 4 °C for 2 h. Beads were washed with PBST lysis buffer three times. Proteins were released from beads after denatured and boiled, then separated with SDS-PAGE for immediate immunoblotting.

### 2.6. Plasmids and Transfection

The EEC were cultured in IMDM Medium (Gibco) containing 10% FBS in six-well plates. The 3T3 cells were cultured in DMEM Medium (Gibco) containing 10% FBS in six-well plates. Overexpression group was transfected with a plvx-Puro vector containing full-length mouse *Rnh1* sequence. RNAi group was transfected with *Rnh1* RNAi targeting to nucleotides 263 to 289 within the *Rnh1* mRNA (GenBank accession no. NM_001172100.1). RT-qPCR analyses evaluated the efficiency of *Rnh1* overexpression or knockdown. The mutant *Rnh1* was constructed by Mut Express II Fast Mutagenesis Kit V2 (Vazyme, Nanjing, China).

EEC or 3T3 cells were transfected with plasmids using HighGene transfection reagent (Abclonal, Wuhan, China) according to the manufacturer’s instructions. Proteins were harvested at 48 h post-transfection.

### 2.7. Cell Treatment

For stress simulation, primary EEC or PC1 cells were treated with 200 µM PA (Macklin, Shanghai, China) or 10 µg/mL of LPS (Sigma-Aldrich), respectively, for 48 h to establish the cell model of oxidative stress or inflammatory damage as reported previously [28,29].

### 2.8. The Construction of the Inflammation Model

In the inflammation model, 8-week-old male mice were injected intraperitoneally with LPS (10 mg/kg) every other day for 1 week as described previously [15]. Control mice were injected with the same volume (100 µL) of sterile saline. All dilutions were made in endotoxin-free 0.9% NaCl (*w*/*v*) water.

### 2.9. RNA Extraction, Reverse Transcription, and Quantitative RT-PCR Analysis

Tissues or cells were homogenized in TRIzol reagent (Invitrogen, Frederick, MD, USA) and total RNA was extracted according to the manufacturer’s protocol. cDNA was synthesized according to the PrimeScript™ RT reagent Kit (Takara, Shiga, Japan) and total cDNA was amplified using TB Green^®^ Premix DimerEraser™ (Takara) on a 7500 real-time PCR system (Applied Biosystems, Foster City, CA, USA). Primer sequences for reverse transcription and RT-qPCR analysis are listed in Supplementary Table S1.

### 2.10. Small RNA qPCR

Tissues or cells were homogenized in TRIzol reagent and the total RNAs were extracted according to the manufacturer’s protocol. For validation of the differential tsRNAs levels, 1 µg RNA was reverse-transcribed using PrimeScript™ RT reagent Kit (Takara) and tsRNA-specific stem-loop RT primers (Supplementary Table S1). RT-qPCR was performed to measure small RNA expression on the ABI ProFlex PCR thermal cycler (Applied Biosystems). U6 was used as an endogenous control to standardize differences.

### 2.11. Extraction and Identification of Exosomes

To isolate exosomes from the cell supernatant, serum-free medium was collected and concentrated by ultrafiltration (3000× *g*, 30 min, 4 °C). Then, polymer precipitation of the concentrated medium was performed by using the Hieff Quick Exosome Isolation Kit (Yeasen), as described previously [30]. Finally, the precipitated exosomes were resuspended in PBS solution and further purified by means of an exosome purification column (Yeasen).

### 2.12. Nuclear and Cytoplasmic Proteins Extraction

Nuclear and cytoplasmic proteins were extracted by Nuclear and Cytoplasmic Protein Extraction Kit (Yeasen). The cells in the Petri dish were washed with PBS three times and scraped off with a cell scraper and blown down into a pipette by centrifugation at 100× *g* for 5 min to collect the cell sediment. Two hundred µL of PMSF-containing Cytoplasmic Extraction Reagent A was added in twenty µL of cell sediment. Cellular precipitate was vortexed vigorously at maximum speed for 5 s to completely suspend. Ten µL of Cytoplasmic Protein Extraction Reagent B was added. The mixture was vortexed vigorously at maximum speed for 5 s and incubated in an ice bath for 1 min. The mixture was vortexed vigorously again at maximum speed for 5 s and centrifuged at 12,000~16,000× *g* for 5 min at 4 °C. Subsequently, the supernatant was immediately pipetted into a pre-cooled plastic tube, and then the supernatant containing cytosolic proteins was extracted. After completing aspiration from the supernatant, 50 µL of nuclear extraction reagent containing PMSF was added to the precipitate. Next, the precipitate was vortexed vigorously at maximum speed for 15~30 s to completely suspend and disperse the precipitate. The mixture was then returned to an ice bath and vortexed vigorously at high speed for 15–30 s every 1–2 min during a total of 30 min. Centrifugation was performed at 12,000~16,000× *g* for 10 min at 4 °C. The supernatant was immediately aspirated into a pre-cooled plastic tube, which contained the extracted nuclear proteins. GAPDH was used as a cytoplasmic reference gene, whereas H3 was used as a nuclear reference gene.

### 2.13. Statistical Analysis

Statistical analyses were performed by GraphPad Prism version 8 software, including an unpaired Student’s *t*-test. All data are expressed as mean ± SD, representing three independent experiments. ns represents not significant; * *p* < 0.05; ** *p* < 0.01; and *** *p* < 0.001.

## 3. Results

### 3.1. RNH1 Is Highly Expressed in Mouse Caput Epididymis

**Figure 1:**
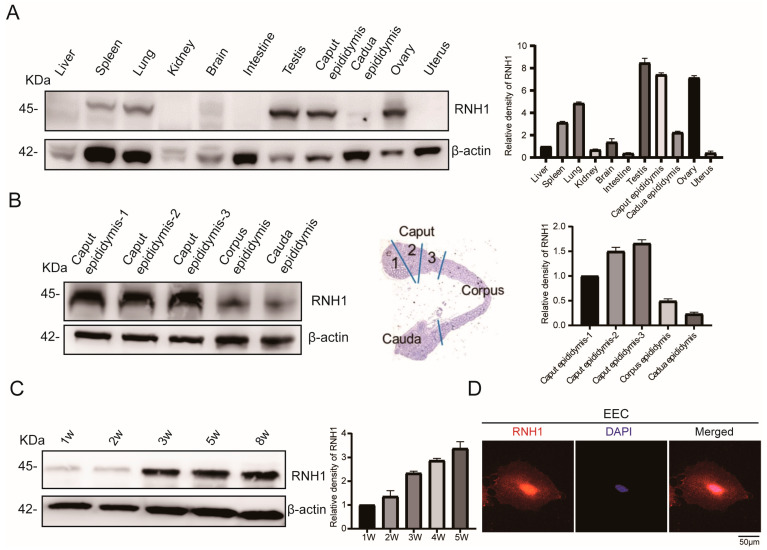
**High RNH1 expression in caput epididymis.** (**A**) Expression of RNH1 protein detected by Western blotting in various mouse tissues. The tissue samples (liver, spleen, lung, kidney, brain, intestine, testis, caput epididymis and cauda epididymis) were from 8-week-old male mice, and ovarian and uterine tissues were from 8-week-old female mice. (**B**) The expression of RNH1 in caput epididymis, corpus epididymis, and cauda epididymis. (**C**) Western blotting detection of RNH1 in caput epididymis at 1 w, 2 w, 3 w, 5 w, and 8 w, respectively. (**D**) Immunofluorescent staining was used to identify localization of RNH1 protein in EEC.

To determine whether RNH1 expression has possible biological function, Western blot analysis was used to compare its protein expression levels in different mouse tissues. The results showed that among these 11 different mouse tissues, RNH1 was more highly expressed in the testis, ovary and caput epididymis than in the liver, spleen, lung, kidney, brain, and intestine (Figure 1A). The high level of RNH1 expression in the epididymis suggested that it may be involved in the reproductive process during sperm epididymal maturation.

The epididymis is a critical reproductive organ involved in sperm maturation, and it has marked importance in the caput epididymis. To explore the role of RNH1 in male reproduction, we measured the RNH1 expression levels in three different sections of the mouse epididymis. They include the caput epididymis, corpus epididymis, and cauda epididymis. Western blotting analysis indicated that the RNH1 expression level was much higher in the mouse caput epididymis than in either the corpus epididymis or the cauda epididymis (Figure 1B). It is noteworthy that the RNH1 protein expression levels were higher in the second and third parts of the caput epididymis (Figure 1B), which is consistent with its role in mediating exosome excretion [10,11]. There are three developmental periods in the postnatal epididymis. They include the undifferentiated period, differentiation period, and post-differentiation period. Western blot analysis showed that the expression level of RNH1 increased in the caput epididymis during its postnatal development. Initially, RNH1 in the mouse caput epididymis were present at a very low level before 3 weeks (corresponding to the undifferentiated period), and then they gradually rose from 3 to 5 weeks (corresponding to the differentiation period); finally, after 8 weeks (corresponding to the post-differentiation period), the RNH1 expression level reached its highest level in the caput epididymis (Figure 1C). Moreover, in order to precisely document the RNH1 distribution in the mouse caput epididymis, we isolated primary epididymal epithelial cells (EEC). The results of immunofluorescence staining revealed that RNH1 in the EEC were distributed in both the nucleus and the cytoplasm (Figure 1D). Therefore, all these results indicated that the function of RNH1 may vary during epididymal epithelial cell development and sperm maturation.

### 3.2. Interaction between RNH1 and ANG in EEC

**Figure 2:**
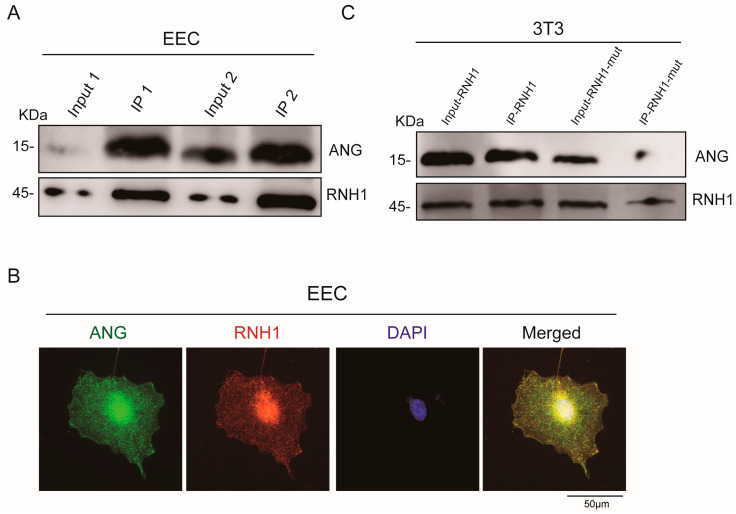
**Interaction between RNH1 and ANG in epididymal epithelial cells.** (**A**) Western blotting analysis validated interaction between RNH1 and ANG in EEC. (**B**) Immunofluorescent staining of ANG (green), RNH1 (red) and DAPI (blue) in EEC. (**C**) Western blotting analysis detected the interaction between the mutant RNH1 and ANG in 3T3 cells.

Several studies showed that RNH1 interacts with ANG in different cell types [31]. To validate this relationship between RNH1 and ANG in EEC, co-immunoprecipitation analysis was performed. Western blotting confirmed an interaction between RNH1 and ANG (Figure 2A), indicating that RNH1 can bind with ANG in the caput epididymis and subsequently RNH1 may block ANG function. Thus, to further investigate the interaction between these two proteins, immunofluorescence staining was also performed in EEC. The results showed that RNH1 and ANG co-localized in both the EEC nucleus and cytoplasm (Figure 2B). Furthermore, a previous study reported that the mutation of Gly85 and Gly86 in the ANG protein disrupted its interaction with RNH1 [17]. Then, we constructed the mutant RNH1 (RNH1-mut), in which W263R and S289R are its sites of interaction with ANG [32]. The co-immunoprecipitation results strongly suggested that the interaction of RNH1 and ANG was largely disrupted in the 3T3 cell line (Figure 2C). Thus, our findings suggested that RNH1 interacted with ANG in the caput epididymis, which may be involved in the generation of tsRNAs.

### 3.3. Upregulated Epididymal Cytoplasmic ANG Levels under Environmental Stress In Vivo and In Vitro

**Figure 3:**
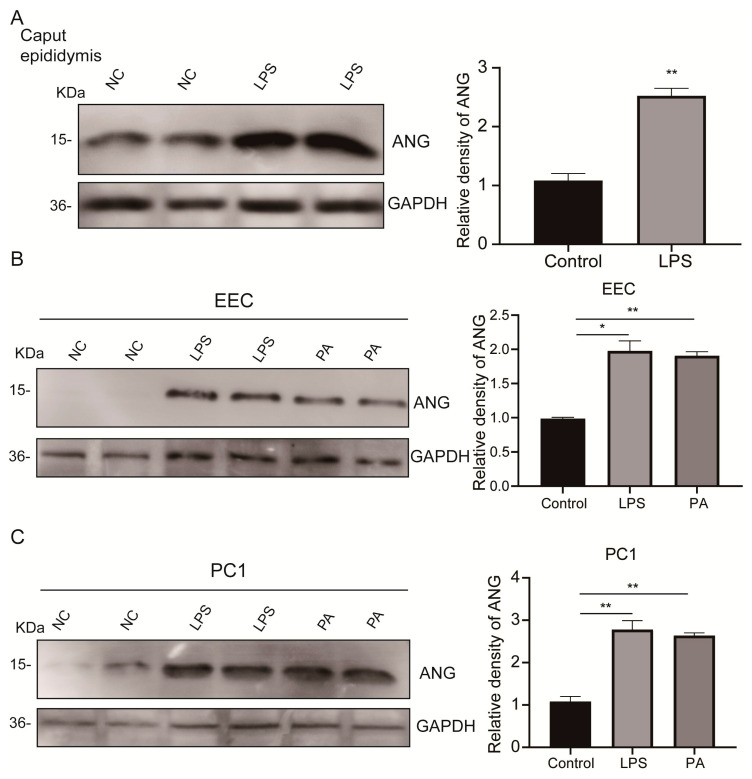
**Environmental stress-induced upregulation of both in vivo epididymal cytoplasmic ANG expression and in vitro.** (**A**) Western blot analysis of ANG in the cytoplasm of caput epididymis identified stress-induced ANG upregulation. (**B**) Western blot analysis of stress-induced upregulation of cytoplasmic ANG expression in EEC after 48 h. (**C**) Western blotting of ANG protein expression in the cytoplasm of PC1 cells after 48 h exposure to stress conditions. Average value of control group was identified as 1. * *p* < 0.05, ** *p* < 0.01.

Inflammation or oxidative stress can induce the expression of ANG [33]. To investigate whether some representative environmental stresses can upregulate the expression levels of ANG in the caput epididymis or EEC, we constructed mouse inflammation models by injecting a pathophysiological dose of lipopolysaccharide (LPS) into the mouse abdominal cavity. Since ANG generates tsRNAs through cleavage of tRNA in the cytoplasm [34], we utilized a cytoplasmic extraction reagent to separate and isolate ANG protein and subsequently measure their expression levels in the cytoplasm of the caput epididymis. Compared to control mice, Western blot analysis showed that the level of cytoplasmic ANG was significantly higher in the caput epididymis isolated from LPS-injected mice (Figure 3A). Moreover, we exposed EEC and proximal caput epididymis-1 (PC1) cells to pathophysiological concentrations of LPS or palmitate (PA). These stressors significantly upregulated ANG expression levels in the cytoplasm of both EEC (Figure 3B) and PC1 cells (Figure 3C). In conclusion, relevant inflammation and oxidative stress upregulated the cytoplasmic ANG expression levels in EEC and PC1 cells, and these alterations may lead to rises in the sperm tsRNAs.

### 3.4. Inflammation and Oxidative Stress Weakened the Interaction of RNH1 and ANG in EEC

**Figure 4:**
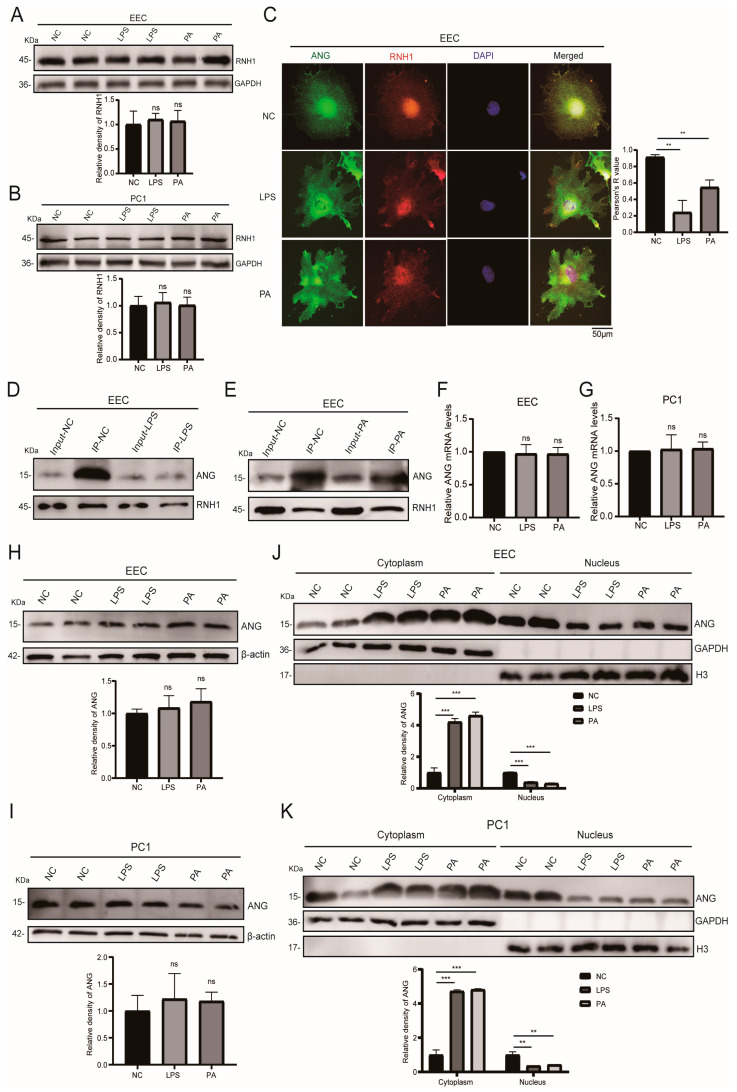
**Inflammation and oxidative stress weaken the interaction between RNH1 and ANG.** (**A**,**B**) Western blot analysis of RNH1 in cytoplasm under environmental stresses in EEC (**A**) and PC1 cells (**B**). (**C**) Immunofluorescent staining reveals localization of RNH1 and ANG under LPS or PA treatment. (**D**,**E**) Western blot analyses documents interaction between RNH1 and ANG under LPS (**D**) and PA (**E**) treatments in EEC. (**F**,**G**) RT-qPCR analysis reveals ANG mRNA levels under LPS or PA treatment in EEC (**F**) and PC1 cells (**G**). (**H**,**I**) Western blot analysis reveals ANG levels in EEC (**H**) and PC1 (**I**), respectively, under LPS or PA treatment. (**J**,**K**) Western blot analysis reveals ANG cytoplasmic and nuclear expression levels in EEC (**J**) and PC1 cells (**K**), respectively, under environmental stresses. GAPDH was used as a cytoplasmic reference gene, whereas H3 was used as a nuclear reference gene. ns: no significance. ** *p* < 0.01, *** *p* < 0.001.

RNH1 is an inflammation and oxidative stress sensor in different types of cells [17,35]. To investigate its role in the epididymis under environmental stress, we measured the RNH1 protein expression level in EEC resulting from either LPS or PA treatment. Western blot analysis showed that the RNH1 expression levels were unchanged under environmental stress in the cytoplasm of EEC (Figure 4A) and PC1 cells (Figure 4B). Moreover, to probe for whether environmental stress can interfere with the interaction of RNH1 and ANG, immunofluorescence staining analysis measured both RNH1 and ANG protein localization in EEC. We found that either LPS or PA treatment weakened their co-localization in both the nucleus and cytoplasm of EEC (Figure 4C). Meanwhile, we discovered that EEC nuclear ANG localization significantly decreased (Figure 4C). Additionally, co-immunoprecipitation analysis was performed in EEC under LPS or PA treatment in order to evaluate the interaction of RNH1 and ANG. Such treatment significantly weakened the interaction between RNH1 and ANG in whole cells (Figure 4D,E).

Since exposure to representative environmental factors increased the cytoplasmic ANG expression levels in EEC, we presumed that this response was mediated through upregulation of its mRNA expression. The results of RT-qPCR analysis showed instead that the mRNA level of ANG was invariant in both EEC (Figure 4F) and PC1 cells (Figure 4G). Western blot analysis also showed that the ANG protein expression levels remained unchanged in whole EEC and PC1 cells (Figure 4H,I). ANG cleaves tRNAs in the cytoplasm in response to various stresses, which then generate tsRNAs and they ultimately inhibit translation initiation [34]. Thus, we hypothesized that environmental stress weakened the interaction of RNH1 and ANG, which resulted in ANG release from the nucleus into the cytoplasm. Therefore, under either LPS or PA treatment, we detected both nuclear and cytoplasmic ANG levels in EEC. Western blot analysis showed these stresses increased the cytoplasmic ANG expression levels, whereas its nuclear expression level decreased in EEC (Figure 4J,K). Accordingly, we conclude that either an inflammatory or oxidative stress weakens the interaction between RNH1 and ANG in the EEC. This detachment unwinds nuclear ANG, which is then translocated to the cytoplasm to finally generate more tsRNAs.

### 3.5. LPS or PA Treatment Increases Levels of tsRNAs in Epididymal Epithelial Cells

**Figure 5:**
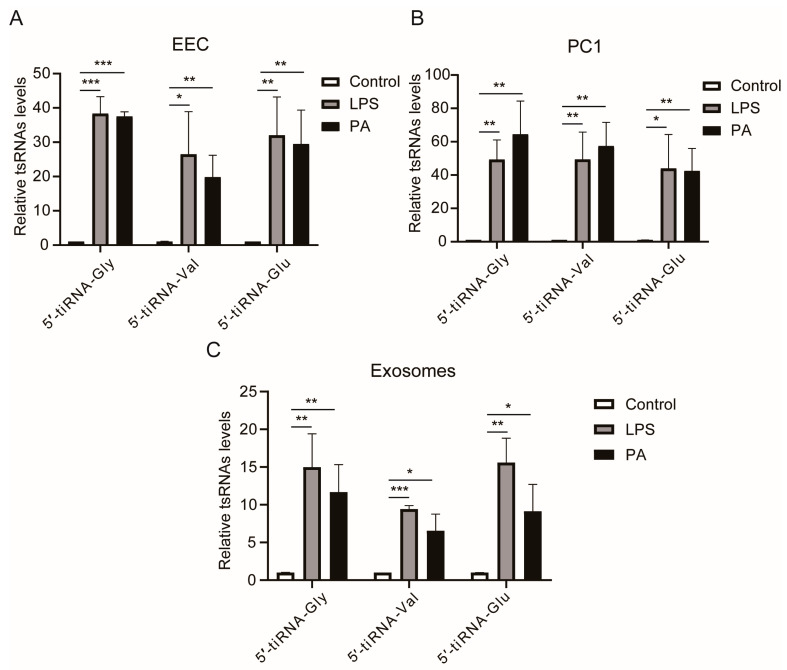
**LPS or PA-induced rises in tsRNAs in EEC and exosomes.** (**A**) The levels of three tsRNAs, 5′-tiRNA-Gly, 5′-tiRNA-Val, and 5′-tiRNA-Glu in EEC under stress conditions. (**B**) RT-qPCR analyses of three tsRNAs in PC1 in the presence of LPS or PA. (**C**) RT-qPCR analyses of three tsRNAs in exosomes isolated from EEC under LPS or PA treatment. * *p* < 0.05, ** *p* < 0.01 and *** *p* < 0.001.

Since inflammation and oxidative stress weakened the interaction between RNH1 and ANG, we determined the underlying mechanism that accounts for how ANG upregulates the production of tsRNAs. This was performed by measuring the expression levels of three tsRNAs. They include 5′-tiRNA-Gly, 5′-tiRNA-Val, and 5′-tiRNA-Glu, which ANG generated under stress [15]. RT-qPCR analysis showed that either LPS or PA treatment increased all their expression levels in EEC (Figure 5A) and PC1 cells (Figure 5B). In addition, the effects of these two stresses were measured on the tsRNA content in exosomes that were released from EEC. As anticipated, their expression levels also increased in both the isolated exosomes and in EEC (Figure 5C). Therefore, we hypothesized that environmental stress exposure induces cytoplasmic increases in ANG expression levels in EEC, which enhances the process of tRNA cleavage and then upregulates the levels of tsRNAs. These rises account for their heightened transfer by exosomes into sperm in the epididymal fluid that EEC secrete.

### 3.6. RNH1 Participates in the Process of tsRNA Generation Produced by ANG in EEC

**Figure 6:**
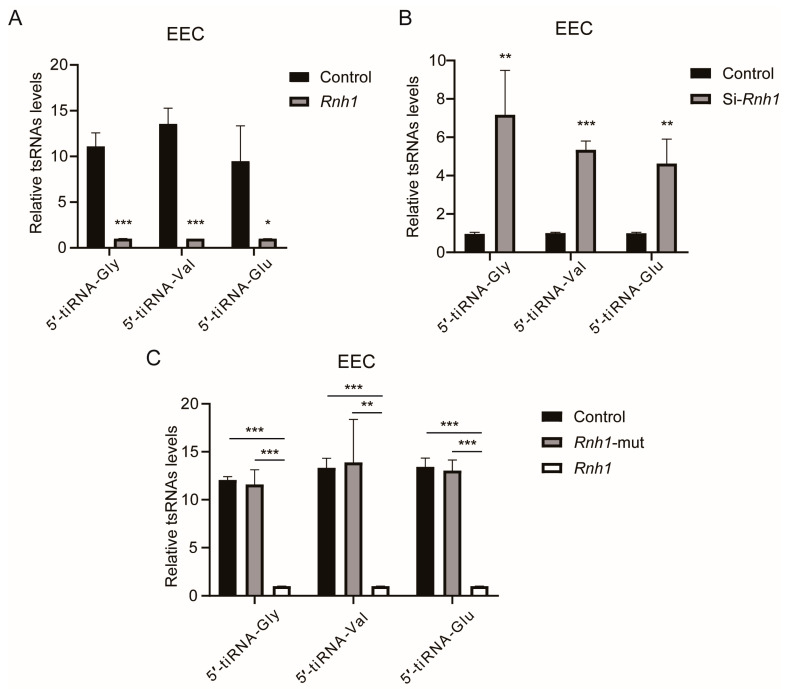
**RNH1 blunts ANG-induced increases in tsRNA generation.** (**A**) RT-qPCR showed that overexpression of *Rnh1* significantly decreased the level of three tsRNAs, 5′-tiRNA-Gly, 5′-tiRNA-Val, and 5′-tiRNA-Glu, in EEC. (**B**) The levels of three tsRNAs increased in *Rnh1* knocked down EEC. (**C**) RT-qPCR analysis of three tsRNA levels in *Rnh1*-overexpressed EEC compared to those in the mutant *Rnh1* transfected EEC or in the control group (vector plasmid transfected EEC). * *p* < 0.05, ** *p* < 0.01 and *** *p* < 0.001.

To validate that RNH1 participates in the process of tRNA cleavage, we overexpressed the *Rnh1* gene in EEC in vitro. The results confirm that the overexpression of *Rnh1* significantly decreased the expression levels of the three abovementioned tsRNAs (5′-tiRNA-Gly, 5′-tiRNA-Val, and 5′-tiRNA-Glu), suggesting that RNH1 plays a critical role in the generation of tsRNAs in mammalian cells (Figure 6A). Conversely, the levels of these three tsRNAs significantly increased when *Rnh1* gene was knocked down in EEC (Figure 6B). Since the linkage sites are known of RNH1 and ANG [32], the constructed mutant *Rnh1* forms were transfected into EEC and the expressed levels of 5′-tiRNA-Gly, 5′-tiRNA-Val, and 5′-tiRNA-Glu were measured. The results also showed that the levels of these three tsRNAs were much higher in the mutant *Rnh1*-transfected EEC and the control group (vector plasmid transfected EEC) than those in the *Rnh1*-overexpressed EEC (Figure 6C). Meanwhile, these three tsRNAs expression levels were the same as those expressed by the mutant *Rnh1*-transfected EEC and the control group. Thus, our findings clearly demonstrate that RNH1 plays an important role in the generation of sperm tsRNAs.

## 4. Discussion

The intergenerational inheritance of acquired phenotypes induced by exposure to representative environmental stresses is a timely research topic generating much interest in the epigenetics research community. The relevance of factors such as maternal health, lifestyle, and perinatal conditions that can result in embryonic diseases is receiving extensive attention in recent years [36,37,38]. However, the possible long-term effects of paternal factors have not been adequately evaluated on embryonic development and offspring health. Increasing evidence documents that unhealthy paternal nutritional status, such as a high-fat, high-sugar, or low-protein diet in mammals, can indeed induce poor sperm quality and non-DNA sequence-based epigenetic changes in sperm, finally leading to metabolic disorders in the offspring [6,39,40].

tsRNAs, a paternal epigenetic factor, have been generally recognized by many different types of studies [7], whereas ANG is an endonuclease which cleaves tRNA in mammalian caput epididymis [15]. On the other hand, in most reports, the heritable tsRNA alterations are mainly and passively caused by exposure to external environmental stressors [41]. Moreover, the underlying mechanism that describes how some environmental factors alter ANG biological function and ultimately induce changes in tsRNA expression levels is rarely studied. In the current study, we found that RNH1, an external environmental sensor, interacts with ANG in epididymal epithelial cells. Furthermore, our results show that RNH1 is involved in the process of tsRNA generation produced by ANG. Therefore, these findings elucidate how environmental factors such as inflammation and oxidative stress initially increase the generation of tsRNAs in the epididymis and ultimately alter paternal inheritance.

tsRNAs vary in lengths between 16 and 35 nucleotides (nts) and are produced by cleavage at different sites on mature tRNAs or precursor tRNAs [42,43], which are precisely regulated by different ribonucleases such as Dicer, ANG, and RNase T2 [22]. According to their origin and composition, these tsRNAs have been grouped into six different classes, including tRF-5s and tRF-3s, i-tRFs, tRF-1, and 5′- and 3′-tRNA halves (also called tiRNAs) [44]. The biogenesis of different tsRNA species is attributed to endonucleases targeting the varied tRNA loop structures (D-, anticodon-, variable-, and T-loops). For instance, the generation of short tsRNAs via the cleavage in the D or T regions occurs in a Dicer-dependent manner [45]. Under hypoxic conditions, Dicer1 can upregulate the expression of tRF-20-MEJB5Y13 [46]. ANG generates tiRNAs by cleaving tRNAs in the anticodon region. This process is triggered by a variety of environmental stresses stimuli, such as oxidation, heat/cold shock and UV irradiation in cancer cells [47,48,49]. Meanwhile, the yeast RNase T2, Rny1p, generates long tsRNAs via the cleavage in the anticodon region [50,51] and it is also the main producer of long and short tsRNAs in plants [52]. The current study employed immunoprecipitation and immunofluorescence analyses to reveal that RNH1 interacts with ANG in the mouse caput epididymis, which is involved in tsRNA generation.

Prior studies showed that exposure to an environmental stress altered sperm tsRNAs. For example, one study initially reported that a subset of tsRNAs in sperm have an altered expression profile and RNA modifications appear in mice fed a high-fat diet [53]. Subsequently, another study demonstrated that even excessive maternal feeding programmed hedonic and metabolic phenotypes across several generations through transmission of sperm tsRNAs into the offspring [37]. Moreover, factors such as a low-protein diet, aged paternal sperm, chemical exposure, and infection can also induce dysfunctional changes in the expression profile of sperm tsRNAs [6,54,55,56]. These altered sperm tsRNAs have been documented to participate in intergenerational epigenetic inheritance of paternal traits [6,7]. In the present study, Western blot analysis showed that the ANG protein expression level was upregulated in the caput epididymis cytoplasm of a murine inflammation model. Meanwhile, as a consequence of either LPS or PA treatment, increases occurred in the ANG protein expression levels in the cytoplasm of EEC and the PC1 cell line, whereas the interaction of RNH1 and ANG was weakened to a large degree in EEC. Furthermore, such stresses instead increased the ANG-induced expression levels of tsRNAs, 5′-tiRNA-Gly, 5′-tiRNA-Val, and 5′-tiRNA-Glu. Accordingly, our results suggest that exposure to environmental stressors such as LPS or PA weaken the binding and interaction between RNH1 and ANG, resulting in the release of ANG from the nucleus and increases in the levels of both ANG and tsRNAs in the cytoplasm of EEC. Additionally, ANG also cleaves pRNA in the nucleolus to de-repress rDNA transcription, activate rRNA production, and subsequently enhance global protein synthesis by increasing ribosome biogenesis [57,58,59]. Interestingly, in recent years, numerous rsRNAs were discovered in sperm by a new spinoff procedure of small RNA sequence analysis such as PANDORA sequencing. These procedures identified altered rsRNAs which also participate in the process of sperm maturation [60].

Notably, several studies have shown that RNH1 is a cytosolic leucine-rich repeat protein that can directly respond to an environmental stress [61]. For instance, under inflammatory conditions, RNH1 inhibits inflammatory vesicle activation by controlling proteasome-mediated degradation of cystathione-1, a downstream effector molecule [35,62]. On the other hand, RNH1 plays an antioxidant role in the protection of cells from redox stress by inhibiting the RNases [16]. To confirm the function of RNH1 on regulating tsRNA generation produced by ANG, we overexpressed the *Rnh1* gene in EEC in vitro. This procedure significantly decreased the expression levels of tsRNAs, 5′-tiRNA-Gly, 5′-tiRNA-Val, and 5′-tiRNA-Glu, which are produced by ANG. Conversely, when we knocked down the *Rnh1* gene in EEC, the levels of these three tsRNAs significantly increased. Additionally, since the interaction sites of RNH1 and ANG have been reported, we then constructed a mutant *Rnh1* to evaluate the interaction between RNH1 and ANG. Western blot analysis showed that interaction between these two proteins was largely disrupted in the mutant transfected *Rnh1*-3T3 cells. Furthermore, the three tsRNA expression levels were much higher in both the mutant transfected *Rnh1*-EEC group and the control group (vector plasmid transfected EEC) than those in the overexpressed *Rnh1*-EEC groups.

It should be pointed out that the aim of the current study entailed determining a potential role of the novel tsRNAs in sperm because a great number of clinical studies demonstrated that several tsRNAs could be new biomarkers in the diagnosis and treatment of diverse types of cancer, such as breast cancer, ovarian cancer, lung cancer, prostate cancer, and colorectal cancer [49,63]. Moreover, one study revealed that some sperm tsRNAs can serve as candidate biomarkers for the diagnostic assessment of sperm epigenetic health and their potential effects on the metabolic health of the offspring [64]. However, a clear mechanism underlying this intergenerational epigenetic inheritance, especially the accurate source and function of these novel tsRNAs during the development and metabolic regulation in the offspring, still needs to be further elucidated. Since tsRNAs are similar to miRNAs in length and both of them have 5′-phosphate and 3′-hydroxyl groups, they can serve as key regulators of myriad biological processes in gene transcription or translation [65,66]. For instance, some reports showed that tsRNAs are important for the maintenance of normal homeostasis, ribosome biogenesis, regulating the cell cycle, retrotransposon regulation, and inhibiting protein synthesis under various conditions [67,68,69]. Therefore, we hypothesized that the tsRNAs regulate the expression of proteins, such as histone deacetylase, histone demethylase, and histone methyltransferase, which participate in the modification of histone. Several recent studies documented that the acquired epigenetic traits could be transmitted to offspring via CpG islands in mice [4,70], and H3K27me3 epialleles of sperm were transgenerationally transmitted in cis locations [71]. Additionally, one report showed that a specific histone modification could also be a transgenerational epigenetic signal relevant to controlling lipid metabolism in *Caenorhabditis elegans* [72].

## 5. Conclusions

**Figure 7:**
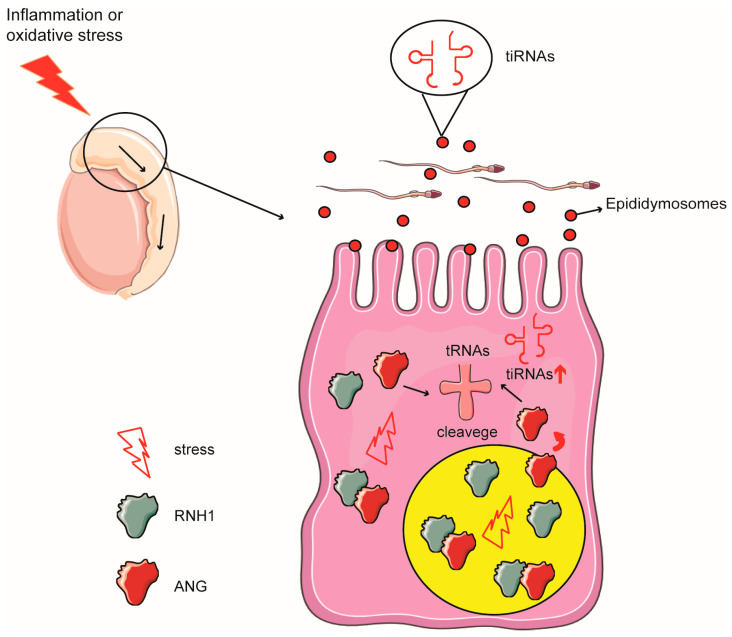
**Schematic representation of RNH1 function in epididymal epithelial cells.** Inflammation and oxidative stress impair RNH1 interaction with ANG in the EEC, which in turn induces ANG release and its translocation from the nucleus to the cytoplasm. Accordingly, rises in the cytoplasmic ANG expression level induce increases in the tsRNA expression levels in both the EEC cytoplasm and its exosomes (epididymosomes). Subsequently, exosomes deliver tsRNAs into the sperm.

Our findings initially demonstrated that RNH1 could be a critical antioxidant in mediating ANG-induced tsRNA biogenesis in mouse caput epididymis. For instance, RNH1 acting as a ribonuclease inhibitor responds to environment factors such as inflammation and oxidative stress and regulates sperm tsRNA generation via its interaction with ANG. Subsequently, the induced acquired traits are transferred to the offspring via exosome delivery in the epididymal fluid (Figure 7). Therefore, as a critical antioxidant to regulate the biogenesis of tsRNAs in epididymis, RNH1 may become a candidate for new markers for paternal intergenerational inheritance. However, additional study is still needed to generate a more detailed mechanism that describes how this type of intergenerational epigenetic inheritance develops. Specifically, it will be relevant to determine how tsRNAs affect control of offspring development; i.e., the relationship still needs to be further investigated regarding how sperm tsRNAs interact with some unique epigenetic factors to control the developmental process.

## Data Availability

Data reported in this paper will be shared by the lead contact upon request. This paper does not report original code. Any additional information required to reanalyze the data reported in this paper is available from the lead contact upon request.

## Supplementary Materials

The following supporting information can be downloaded at: https://www.mdpi.com/article/10.3390/antiox13081020/s1, Supplementary Table S1. Sequences of primers used for RT-qPCR analysis.
